# Sulbactam protects neurons against double neurotoxicity of amyloid beta and glutamate load by upregulating glial glutamate transporter 1

**DOI:** 10.1038/s41420-024-01827-5

**Published:** 2024-02-06

**Authors:** Li Li, Wenbin Li, Wei Jiang, Renhao Xu

**Affiliations:** 1 The Central Laboratory, The Second Hospital of Hebei Medical University, Shijiazhuang, Hebei China; 2 Department of Pathophysiology, Hebei Medical University, Shijiazhuang, Hebei China; 3 Hebei Collaborative Innovation Center for Cardio-Cerebrovascular Disease, Hebei Vascular Homeostasis Key Laboratory, The Second Hospital of Hebei Medical University, Shijiazhuang, Hebei China

**Keywords:** Diseases, Neuroscience

## Abstract

Amyloid beta (Abeta) synergistically enhances excitotoxicity of glutamate load by impairing glutamate transporter 1 (GLT1) expression and function, which exacerbates the development of Alzheimer’s disease (AD). Our previous studies suggested that sulbactam can upregulate the expression levels and capacity of GLT1. Therefore, this study aims to investigate whether sulbactam improves neuronal tolerance against neurotoxicity of Abeta and glutamate load by up-regulating GLT1 in primary neuron-astrocyte co-cultures. Early postnatal P0–P1 Wistar rat pups’ cortices were collected for primary neuron–astrocyte cultures. Hoechst–propidium iodide (HO–PI) stain and lactate dehydrogenase (LDH) assays were used to analyze neuronal death. Cell counting kit 8 (CCK8) was applied to determine cell viability. Immunofluorescence staining and western blotting were used to assess protein expressions including GLT1, B-cell lymphoma 2 (BCL2), BCL2 associated X (BAX), and cleaved caspase 3 (CCP3). Under the double effect of Abeta and glutamate load, more neurons were lost than that induced by Abeta or glutamate alone, shown as decreased cell viability, increased LDH concentration in the cultural medium, HO–PI positive stains, high CCP3 expression, and high BAX/BCL2 ratio resulting from increased BAX and decreased BCL2 expressions. Notably, pre-incubation with sulbactam significantly attenuated the neuronal loss and activation of apoptosis induced by both Abeta and glutamate in a dose-dependent manner. Simultaneously, both astrocytic and neuronal GLT1 expressions were upregulated after sulbactam incubation. Taken together, it could be concluded that sulbactam protected neurons against double neurotoxicity of Abeta and glutamate load by upregulating GLT1 expression. The conclusion provides evidence for potential intervention using sulbactam in AD research.

## Introduction

The removal of glutamate in the synaptic cleft is dependent on glutamate uptake by excitatory amino acid transporters (EAATs), as there is no enzyme metabolizing glutamate in the synaptic cleft. EAAT2, also known as Glutamate Transporter 1 (GLT1), mainly expresses on astrocytes and is responsible for more than 80% of glutamate reuptake in the brain, and thus plays an essential role in the maintenance of glutamate homeostasis and the prevention of glutamate excitotoxicity, which is involved in neuronal damage in many pathological processes [1–3]. Besides the Abeta neurotoxic cascade, many studies indicated GLT1 impairments in the pathogenesis of AD. For example, the autopsy of AD patients revealed decreased GLT1 expression at gene and protein levels in the hippocampus and medial frontal lobe [4–6]. There was aberrant insoluble EAAT2 accumulating in AD [7]. In the APP23 transgenic mouse model of AD, GLT1 expression and glutamate uptake significantly decreased [8, 9]. The loss of GLT1 in APPswe/PS1ΔE9 mice accelerated the development of cognitive deficits and restored EAAT2 function could improve synaptic impairments, Abeta deposition, and cognitive deficiency in AD animal models [10–12]. These reports revealed that the synergistic damage of the neurotoxic cascade of Abeta and excitotoxicity of glutamate load is one of the mechanisms that deteriorate the progression of AD. Therefore, improving GLT1 damage is a new target to inhibit the progression of AD.

Ceftriaxone, a bata-lactam antibiotic, can upregulate GLT1 expression and uptake activity for glutamate and thus protects neurons from ischemic stimulation [13–16]. Our recent study showed that ceftriaxone played a role in improving cognitive disorders, maintaining homeostasis glutamate as a neurotransmitter, and ameliorating synapse loss in the APP/PS1 mouse model of AD [17–22]. However, long-term usage of ceftriaxone will cause side effects, such as bacterial resistance and dysbacteriosis, because of its powerful anti-bacterial effect. These side effects limit its clinical translational study as an anti-AD drug.

Sulbactam is an atypical beta-lactam antibiotic and has no antibacterial effect using alone. Our recent studies showed that sulbactam can upregulate GLT1 expression, increase glutamate uptake, and thus exert anti-cerebral ischemic effects as well [23, 24]. Based on the dysregulation of GLT1 in AD and the upregulating effect of sulbactam on GLT1, it may provide insights and clues for anti-AD research to study the improving effect of sulbactam on GLT1 and its neuronal protection in AD. Therefore, this study, using a soluble Abeta-induced AD model in vitro, investigates the improving effect of sulbactam on neuronal tolerance against glutamate load in the presence of Abeta, and the role of GLT1 expression in the process. Furthermore, the expressions of apoptotic-associated proteins including BCL2, BAX, and CCP3 were examined to investigate the mechanism involved in the process.

## Result

### Neuronal survival

**Fig. 1:**
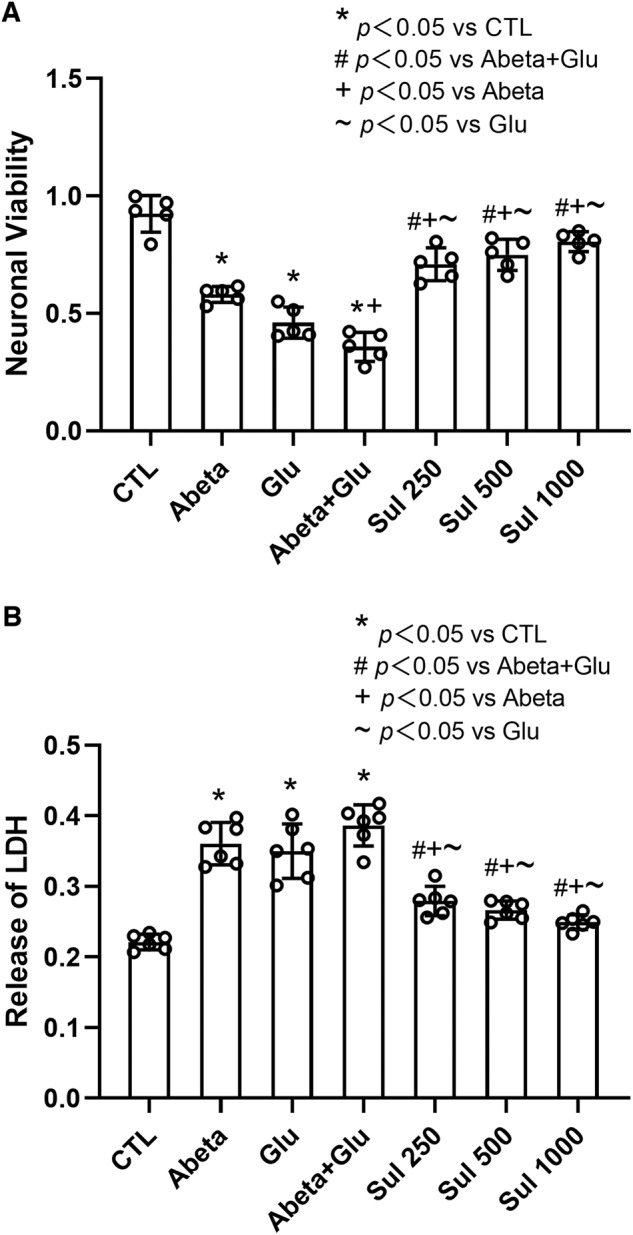
Sulbactam enhanced cell viability and reduced LDH release under the double effect of Abeta and glutamate load in neuron-astrocyte co-cultures. Cell viability (**A**) was determined by CCK8 assay (*n* = 5), and LDH release (**B**) was detected by lactate dehydrogenase assay (*n* = 6). *p < 0.05 vs. CTL, # *p* < 0.05 vs. Abeta + Glu, +*p* < 0.05 vs. Abeta, ~*p* < 0.05 vs. Glu.

The conditions of neuronal survival after the effect of Abeta and/or glutamate load were examined using CCK8, LDH assays, and HO-PI double stains. CCK8 examination showed that the cell viability was significantly decreased after cells were incubated with Abeta by 37% (*p* < 0.0001) and Glu by 50% (*p* < 0.0001) compared to the control group. The double effect of Abeta and glutamate further decreased the cell viability by 61% (*p* < 0.0001) in the Abeta + Glu group compared to the control group. Sulbactam incubation significantly increased the cell viability subjected to both Abeta and glutamate effect in a dose-dependent manner, represented by an increase of 98% in Sul 250 μmol/L (*p* < 0.0001), 110% in Sul 500 μmol/L (*p* < 0.0001), and 125% in Sul 1000 μmol/L (*p* < 0.0001) in Sul + Abeta + Glu group compared to Abeta + Glu group (Fig. 1A).

The concentration of LDH in the culture medium significantly increased after cells were incubated with Abeta by 63% (*p* < 0.0001), Glu by 58% (*p* < 0.0001), and Abeta + Glu by 75% (*p* < 0.0001) compared to the control group. After pre-incubation with sulbactam, LDH release was significantly reduced by 28% (*p* < 0.0001) in Sul 250 μmol/L, 31% (*p* < 0.0001) in Sul 500 μmol/L, and 35% (*p* < 0.0001) in Sul 1000 μmol/L in Sul + Abata + Glu group compared to Abeta + Glu group (Fig. 1B).

**Fig. 2:**
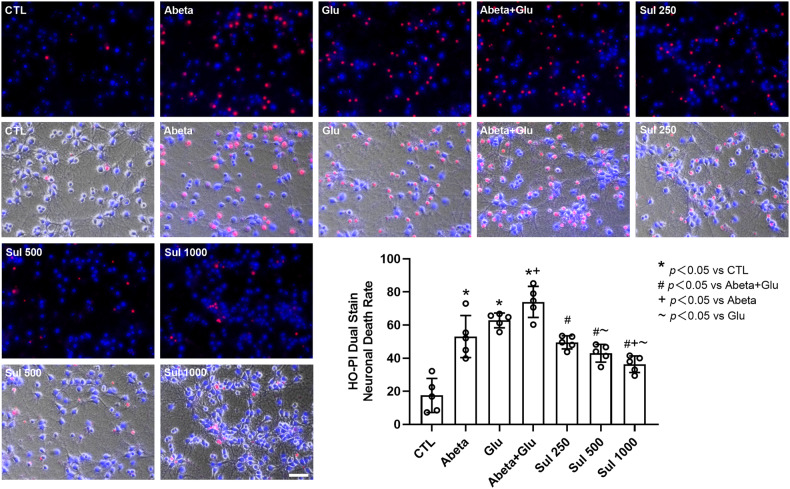
Sulbactam reduced neuronal death induced by the double effect of Abeta and glutamate in neuron-astrocyte co-cultures. The images are representative fluorescent photomicrographs with HO-PI double stains, in which the uppers are fluorescence, and the lower are bright field photomicrographs. The scale bar represents 50 μm. The bar graph in the lower-right corner shows the neuronal death rate under the HO–PI double stains in each group. *n* = 5. *p < 0.05 vs. CTL, # *p* < 0.05 vs. Abeta + Glu, ~*p* < 0.05 vs. Glu.

HO-PI double stains (Fig. 2) showed that after cells were incubated with Abeta, glutamate, and Abeta + glutamate, the number of dead cells increased significantly by 203% (*p* < 0.0001) in Abeta, 259% (*p* < 0.0001) in Glu and 323% (*p* < 0.0001) in Abeta + Glu groups compared to the control group. Pre-incubation with sulbactam significantly reduced the cell death induced by the double effect of Abeta and glutamate by 33% (*p* = 0.0008) in Sul 250 μmol/L, 42% (*p* < 0.0001) in Sul 500 μmol/L, and 51% (*p* < 0.0001) in Sul 1000 μmol/L in Sul + Abeta + Glu group compared to Abeta + Glu group. These results showed the dose-dependency of sulbactam in inhibiting cell death induced by the double effect of Abata and glutamate load.

### BAX/BCL2 ratio

**Fig. 3:**
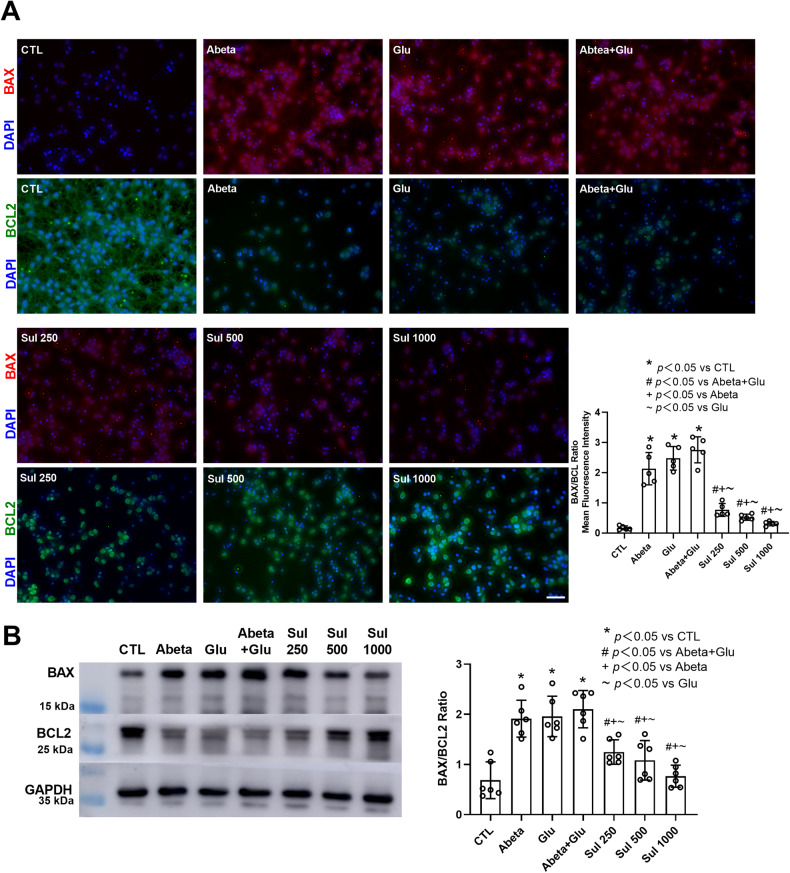
Sulbactam inhibited BAX, while increased BCL2 expressions induced by the double effect of Abeta and glutamate in neuron-astrocyte co-cultures. The uppers are representative immunofluorescent photomicrographs in each group (**A**). The scale bar represents 50 μm. The bar graph in the lower-right corner shows changes in BAX/BCL2 ratios in mean immunofluorescence intensity in each group, *n* = 5. The bottom is western blotting analysis (**B**), in which the left is representative immunoblot bands in each group, and the right bar graph is the quantitative representation of immunoblot bands by the ratios of the integrated density of BAX to BCL2, *n* = 6. *p < 0.05 vs. CTL, #*p* < 0.05 vs. Abeta + Glu, +*p* < 0.05 vs. Abeta, and ~*p* < 0.05 vs. Glu.

The immunofluorescence staining (Fig. 3A) showed that both BAX and BCL2 were distributed in the cytoplasm. Compared to the control group, BAX was highly labeled in Abeta, Glu, and Abeta + Glu groups, while BCL2 immunofluorescent intensity was decreased in these groups. The ratio of BAX/BCL2 immunofluorescent intensity significantly increased by 1162% (*p* = 0.012) in the Abeta group, 1368% (*p* = 0.02) in the Glu group, and 1533% (*p* = 0.018) in Abeta + Glu group compared to the control group. In comparison with the Abeta + Glu group, BAX was decreased and BCL2 was increased after sulbactam pre-incubation in Sul + Abeta + Glu group. The BAX/BCL2 ratio was reduced by 72% (*p* = 0.0012) in Sul 250 μmol/L, 81% (*p* = 0.0034) in Sul 500 μmol/L and 89% (*p* = 0.0023) in Sul 1000 μmol/L in Sul + Abeta + Glu group compared to Abeta + Glu group.

Western blotting analysis (Fig. 3B) showed that compared to the control group, BAX was highly expressed in Abeta, Glu, and Abeta + Glu groups, while BCL2 was less seen in these groups. The ratio of BAX/BCL2 immunoblot intensity significantly increased by 179% (*p* < 0.0001) in the Abeta group, 185% (*p* < 0.0001) in the Glu group, and 206% (*p* < 0.0001) in the Abeta + Glu group compared to the control group. Sulbactam pre-incubation in the Sul + Abeta + Glu group significantly decreased BAX expression and increased BCL2 expression compared to the Abeta + Glu group. The decrease in the BAX/BCL2 ratio was 41% (*p* = 0.0023) in Sul 250 μmol/L, 48% (*p* = 0.0002) in Sul 500 μmol/L and 64% (*p* < 0.0001) in Sul 1000 μmol/L in Sul + Abeta + Glu group compared to Abeta + Glu group.

### CCP3 expression

**Fig. 4:**
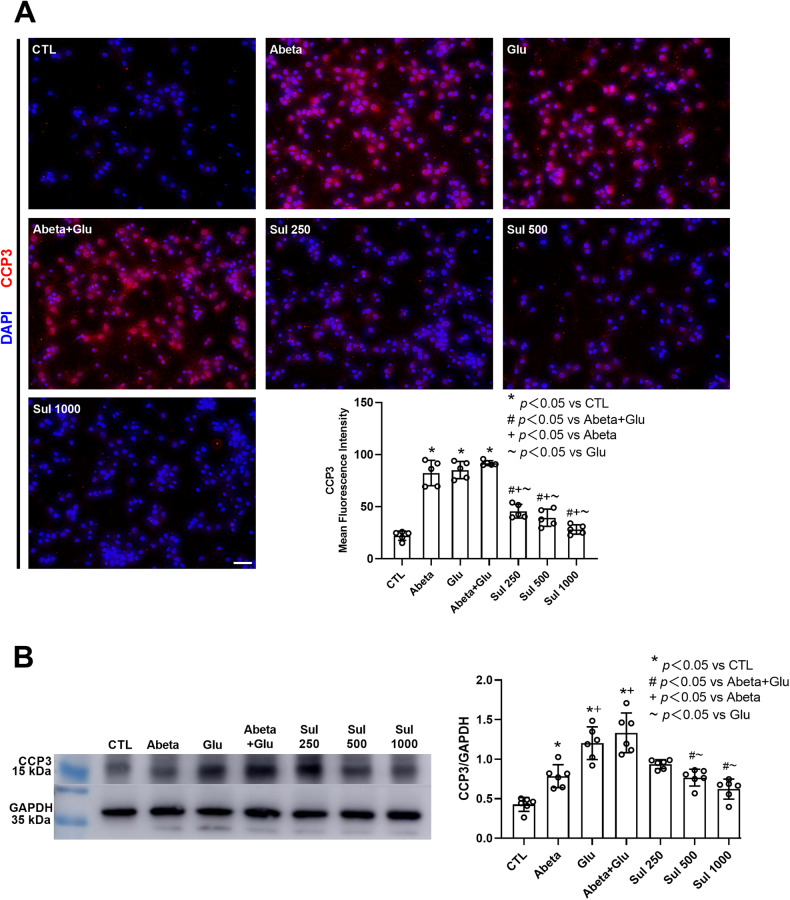
Sulbactam inhibited CCP3 expression induced by the double effect of Abeta and glutamate in neuron-astrocyte co-cultures. The uppers are representative of the immunofluorescent photomicrograph in each group (**A**). The scale bar represents 50 μm. The bar graph in the lower-right corner shows changes in mean immunofluorescence intensity in each group, *n* = 5. The bottom is western blotting analysis (**B**), in which the left is representative immunoblot bands in each group, and the right bar graph is the quantitative representation of the immunoblot bands by the ratios of the integrated density of CCP3 to GADPH, *n* = 6. *p < 0.05 vs. CTL, #*p* < 0.05 vs. Abeta+Glu, +*p* < 0.05 vs. Abeta, ~*p* < 0.05 vs. Glu.

The immunofluorescence staining (Fig. 4A) showed that CCP3 could be labeled in the cytoplasm. Compared to the control group, CCP3 was intensively expressed in the Abeta, Glu, and Abeta + Glu groups, with an increase of 271% (*p* < 0.0001) in the Abeta group, 284% (*p* < 0.0001) in the Glu group and 314% (*p* < 0.0001) in Abeta + Glu group compared to the control group. The intensity of CCP3-positive stain decreased in sulbactam pre-treated groups by 50% (*p* < 0.0001) in Sul 250 μmol/L, 57% (*p* < 0.0001) in Sul 500 μmol/L and 69% (*p* < 0.0001) in Sul 1000 μmol/L in Sul+Abeta + Glu group compared to Abeta + Glu group.

Western blotting analysis (Fig. 4B) showed that compared to the control group, CCP3 was extremely manifested in the Abeta, Glu, and Abeta + Glu groups. Compared to the control group, CCP3 expression significantly increased by 67% (*p* = 0.01) in the Abeta group, 125% (*p* = 0.001) in the Glu group, and 129% (*p* = 0.002) in the Abeta + Glu group. Sulbactam pre-incubation significantly decreased CCP3 expression by 19% (*p* = 0.1) in Sul 250 μmol/L, 30% (*p* = 0.019) in Sul 500 μmol/L, and 39% (*p* = 0.006) in Sul 1000 μmol/L in Sul + Abeta + Glu group compared to Abeta + Glu group.

### GLT1 expression

**Fig. 5:**
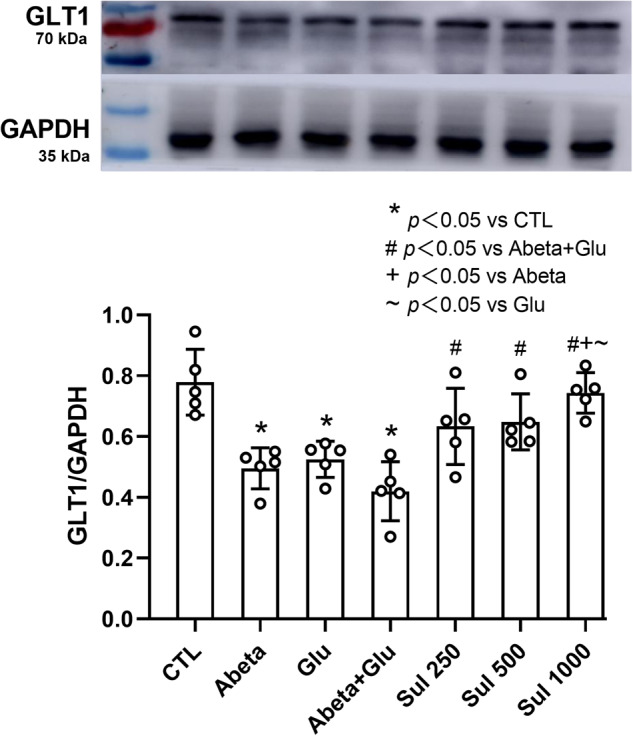
Western blotting analysis shows that sulbactam inhibited GLT1 downregulation induced by the double effect of Abeta and glutamate in primary neuron-astrocyte co-cultures. The upper is representative of immunoblot bands, and the lower bar graph is a quantitative representation of the immunoblot bands by the ratios of the integrated density of GLT1 to GADPH. *n* = 5. *p < 0.05 vs. CTL, #*p* < 0.05 vs. Abeta + Glu, +*p* < 0.05 vs. Abeta, ~*p* < 0.05 vs. Glu.

We first examined the alteration of total GLT1 protein expression in neuron-astrocyte co-cultures. Western blotting analysis (Fig. 5) showed that compared to the control group, GLT1 expression decreased by 36% (*p* = 0.0006) in the Abeta group, 33% (*p* = 0.0023) in the Glu group, and 46% (*p* < 0.0001) in Abeta + Glu group. Sulbactam pre-incubation significantly increased the GLT1 expression in a dose-dependent manner, the increase was 51% (*p* = 0.01) in Sul 250 μmol/L, 54% (*p* = 0.007) in Sul 500 μmol/L, and 77% (*p* < 0.0001) in Sul 1000 μmol/L in Sul + Abeta + Glu group compared to Abeta + Glu group.

**Fig. 6:**
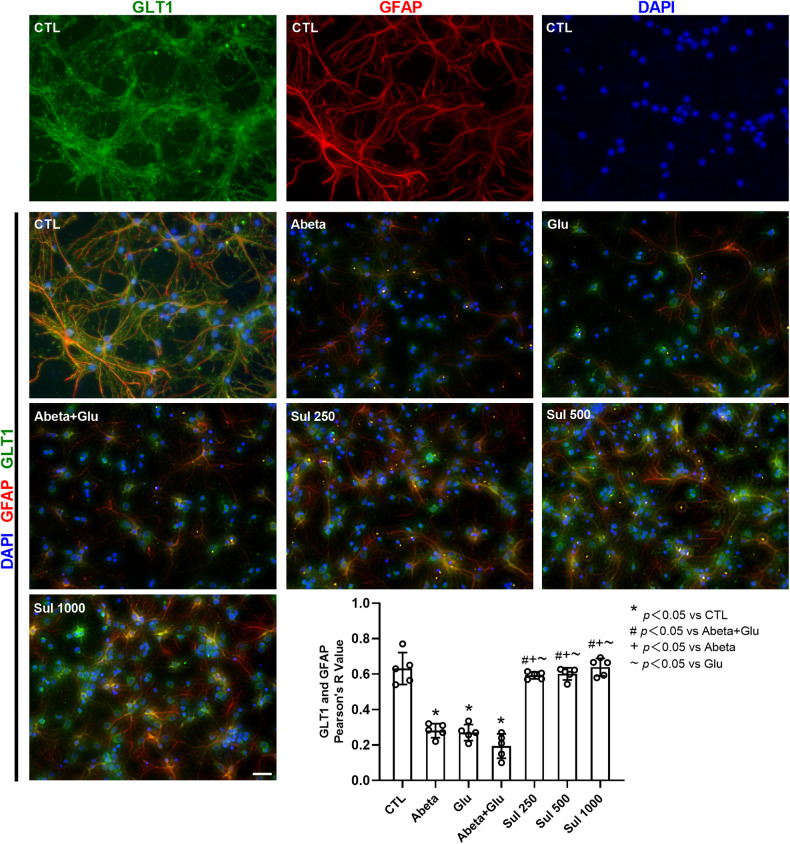
Double immunofluorescence staining of GLT1 and GFAP shows that sulbactam inhibited GLT1 downregulation in astrocytes in primary neuron-astrocyte co-cultures. The photomicrographs in the first line show individual expression of GLT1, GFAP (an astrocyte marker), and DAPI in the control group. Others are merged photomicrographs of GLT1, GFAP, and DAPI. The scale bar represents 50 μm. The bar graph in the lower-right corner shows Pearson’s *R*-value (values range from 1 to −1, 1 for a perfect correlation, 0 for no correlation, and −1 for a perfect anti-correlation) of merged photomicrographs in each group. The co-localization of GLT1 and GFAP was decreased after the incubation of Abeta and glutamate, and sulbactam treatment significantly ameliorated the decrease in co-localization. *n* = 5. *p < 0.05 vs. CTL, # *p* < 0.05 vs. Abeta + Glu, +*p* < 0.05 vs. Abeta, ~*p* < 0.05 vs. Glu.

Next, we investigated the co-localization of GLT1 with GFAP (an astrocyte marker) in neuron-astrocyte co-cultures (Fig. 6). Immunofluorescence staining showed abundant co-localization of GLT1 with GFAP in the control group, indicating the expression of GLT1 in astrocytes. This finding is consistent with previous reports that GLT1 is mainly expressed in astrocytes [25, 26]. The co-localization was reduced by 55% (*p* < 0.0001) in the Abeta group, 57% (*p* < 0.0001) in the Glu group, and 69% (*p* < 0.0001) in the Abeta + Glu group compared to the control group. Sulbactam pre-incubation significantly increased the co-localization by 206% (*p* < 0.0001) in Sul 250 μmol/L, 210% (*p* < 0.0001) in Sul 500 μmol/L, and 230% (*p* < 0.0001) in Sul 1000 μmol/L in Sul + Abeta + Glu groups compared to Abeta + Glu group. These findings suggested that sulbactam upregulated GLT1 expression in astrocytes.

**Fig. 7:**
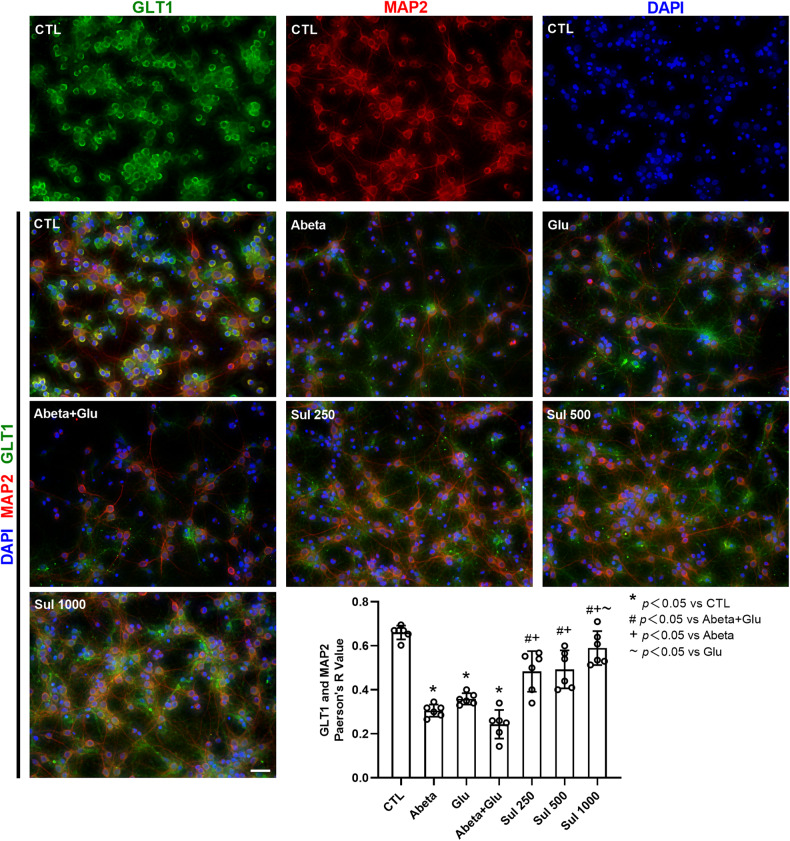
Double immunofluorescence staining of GLT1 and MAP2 shows that sulbactam inhibited GLT1 downregulation in neurons in primary neuron-astrocyte co-cultures. The photomicrographs in the first line show individual expression of GLT1, MAP2 (a marker of the neuron), and DAPI in the control group. Others are merged photomicrographs of GLT1, MAP2, and DAPI. The scale bar represents 50 μm. The bar graph in the lower-right corner shows Pearson’s *R* value (values range from 1 to −1, 1 for a perfect correlation, 0 for no correlation, and −1 for a perfect anti-correlation) of merged photomicrographs in each group. The co-localization of GLT1 and MAP2 was decreased after the incubation of Abeta and glutamate, and sulbactam pre-incubation significantly ameliorated the decrease in co-localization. *n* = 6. *p < 0.05 vs. CTL, #*p* < 0.05 vs. Abeta + Glu, +*p* < 0.05 vs. Abeta, ~*p* < 0.05 vs. Glu.

Lastly, we examined the co-localization of GLT1 with MAP2 (a neuron marker) (Fig. 7). The immunofluorescence staining showed that there were also relatively abundant co-localizations of GLT1 with MAP2 in the control group. The co-localization was reduced by 54% (*p* < 0.0001) in the Abeta group, 46% (*p* < 0.0001) in the Glu group, and 63% (*p* < 0.0001) in Abeta + Glu group compared to the control group. Sulbactam pre-incubation prevented the reduction of co-localization induced by Abeta, glutamate and Abeta+glutamate, shown as an increase by 99% (*p* = 0.0093) in Sul 250 μmol/L, 102% (*p* = 0.0052) in Sul 500 μmol/L and 142% (*p* = 0.0001) in Sul 1000 μmol/L in Sul + Abeta + Glu groups compared to Abeta + Glu group. These findings suggested that sulbactam upregulated GLT1 expression in neurons as well.

## Discussion

The present study revealed that the double effect of Abeta and glutamate load exacerbates neuron loss in neuron-astrocyte co-cultures, and sulbactam strengthens the neuronal tolerance against the double neurotoxicity of Abeta and glutamate load by upregulating GLT1 expression.

Firstly, the present study showed that, under the double effect of Abeta and glutamate, more neurons were lost in neuron-astrocyte co-cultures. Abeta is an important mediator in the pathogenesis of AD. It has been reported that Abeta-induced neurotoxicity cascade can interrupt glutamate uptake of astrocytes, and cause glutamate accumulation in extracellular space [27, 28]. For example, exogenous soluble Abeta reduced GLT1 expression levels and declined glutamate uptake activity in primary cultured astrocytes from rats and mice [29–31]. Administration of Abeta to neuron-astrocyte co-cultures induced glutamate release and resulted in synaptic loss [32, 33]. Abeta 1–42 slowed the clearance of synaptically released glutamate by mislocalizing GLT1 [30]. These findings suggested that Abeta may exacerbate the excito-neurotoxicity of over-accumulated glutamate due to the damage of GLT1 in the pathogenesis of AD. In addition, Abeta can interact with and activate the excitatory amino acid receptor NMDA [27], which may be also involved in more neuronal death under the double effect of Abeta and glutamate overload, because NMDA receptors are important targets mediating the excito-neurotoxicity of glutamate overload [34, 35].

This research demonstrated that necrosis and apoptosis were involved in the mechanisms of neuronal death under the double effect of Abeta and glutamate overload. The increased levels of LDH and numbers of PI-positive and intensive HO-positive cells showed the presence of necrosis in the co-cultures after the treatment of Abeta and/or glutamate. Particularly, the expression of the pro-apoptotic protein BAX upregulated, while the anti-apoptotic protein BCL2 downregulated, which resulted in a significant elevation of BAX/BCL2 ratio, under the double effect of Abata and glutamate. In addition, the expression of another pro-apoptotic protein CCP3 upregulated significantly as well. These changes suggested the involvement of apoptosis under the double effect of Abeta and glutamate load. Parallel results also demonstrated that Abeta 1–42 [36–38] and glutamate [38–42] induced apoptosis in neuronal SH-SY5H cells and primary cultured neurons, which manifested as increased BAX/BCL2 ratio and expression of CCP3. Together, these findings showed that neuronal apoptosis and necrosis were involved in the process under the double effects of Abeta and glutamate load.

Notably, the present study revealed that sulbactam attenuated neuronal death under the double effect of Abeta and glutamate load. Simultaneously, the expressions of apoptosis-related proteins were downregulated after the pre-treatment of sulbactam, suggesting the neuronal protection of sulbactam by inhibiting apoptosis against the double effect of Abeta and glutamate.

Particularly, this research reported that sulbactam significantly upregulated GLT1 expression along with the inhibition of neuronal death induced by exogenous Abeta and glutamate. Firstly, western blotting analysis showed an increase in total GLT1 protein expression. Furthermore, double immunofluorescence staining showed that GLT1 expression in astrocytes in the neuron-astrocyte co-cultures significantly upregulated after the sulbactam pre-incubation. This finding is consistent with previous reports that GLT1 primarily expresses on astrocytes [25, 26], and sulbactam could dose-dependently upregulate GLT1 expression [23, 24, 43, 44]. In addition, the present study also showed that GLT1 co-localized abundantly with neuronal marker MAP2 in the neuron-astrocyte co-cultures after sulbactam treatment, which meant that GLT1 expressed on neurons and its expression was upregulated by sulbactam as well. Previous studies reported that there is a small number of GLT1 expression in neurons besides astrocytes [45, 46], which supports our findings. The relatively abundant GLT1 expression in neurons might be due to that the number of astrocytes in neuron-astrocyte co-cultures was relatively less than that in the brain, leading to the result that GLT1 expression on neurons seemed abundant in neuron-astrocyte co-cultures. Although we did not design a blocking experiment for GLT1 to determine the role of GLT1 in the neuronal protection of sulbactam in the present study, our previous study demonstrated that dihydrokainic acid, a specific blocker of GLT1 activity, inhibited the neuronal protective effect of sulbactam against ischemic stimulation [24], which indicated the essential role of GLT1 in neuronal protection of sulbactam.

Together, our findings suggested, for the first time, that sulbactam protected neurons against double damage of Abeta and glutamate load by upregulating GLT1 expression in astrocytes and neurons. During the development of AD, Abeta triggers the excito-neurotoxicity of glutamate by damaging GLT1 expression and function, and these changes concurrently exacerbate the development of AD [27, 28]. Therefore, the present findings may be particularly significant for the study of the prevention and treatment of AD from multi-facets. Furthermore, given that sulbactam has no antibacterial effect when used alone, and has no side effects such as bacterial resistance or dysbacteriosis, the present findings provide a potential for sulbactam in translational studies of anti-AD.

## Materials and methods

### Primary cell cultures

A litter of early postnatal P0–P1 Wistar rat pups’ cortices were used for neuron-astrocyte co-cultures. All animal experiments were performed in compliance with ARRIVE guidelines and all animal protocols were approved by the Laboratory Animal Welfare and Ethical Committee of the Second Hospital of Hebei Medical University. The cultures were prepared according to the previous report [24]. Briefly, cerebral cortices were isolated and dissociated by 2 mg/ml papain and 2 mg/ml deoxyribonuclease (Pro. No. DN25, Sigma, USA) for 30 min at 37 °C. To terminate enzyme activity, the tissue was rinsed with DMEM-F12 (Cat. No. 11320033, Gibco, China) containing 10% FBS (Cat. No. 10099141C, Gibco, China) twice gently. Cortices were carefully triturated with a fire-polished pipette ten times in a plating medium to dissociate cells. Cells were plated at 500,000/ml onto 24-well or six-well dishes which were pre-coated with 0.1 mg/ml poly-l-lysin and incubated at 37 °C with a gas mixture containing 5% CO_2_ and 95% O_2_. After 3-h incubation, the medium was replaced completely with neurobasal-A (Cat. No. 10888022, Gibco, China) maintenance medium containing 2% B27. Thereafter, the medium was refreshed in half amount every three days. After pre-cultures for 7–9 days, healthy cultures were used for the study.

### Reagent preparation

Abeta 1–42 (Abeta, Cat. No. HY-P1363, MCE, China) was suspended in hexafluoro propanol (HFIP, Pro. No. 105228, Sigma, USA) to an initial concentration of 1 mmol/L on ice. The suspension was incubated at room temperature for one hour and then was aliquoted. HFIP was allowed to evaporate overnight in the hood. The dried peptide film was resuspended in DMSO (Pro. No. D2650, Sigma, USA) to a stock concentration of 5 mmol/L. Before administration, the oligomers were diluted in cold PBS to the concentration of 100 μmol/L. The solution was incubated at 4 °C for 24-h and centrifuged at 14,000×*g* for 10 min at 4 °C to remove insoluble aggregates. The supernatant was soluble Abeta oligomers.

Previous studies showed the neurotoxicity of Abeta at a wide range of concentrations from 2 to 10 μmol/L in rat and human primary hippocampal neuron cultures [47, 48]. According to the studies and our preliminary experiments, we set the final working concentration of Abeta at 10 μmol/L in our cultural system.

Glutamate (Pro. No. G5889, Sigma, USA) was dissolved in sterile PBS to stock solutions at a concentration of 30 mmol/L. Previous studies demonstrated that glutamate at different concentrations ranging from 30 to 100 μmol/L can induce neurotoxicity to primary cultures of cortical neurons [49–51]. Based on the reports and our preliminary experiments, we used glutamate concentration at 60 μmol/L in our cultural system, which reached an overload level to the primary neurons.

Sulbactam sodium (Cat. No. HY-B0334A, MCE, China) was dissolved in sterile PBS to stock solutions at a concentration of 200 mmol/L. The final working concentration of sulbactam was determined at 250, 500, and 1000 µmol/L in our cultural system based on the dosage used in mice [52, 53].

### Grouping and treatments

Cells were divided into control (CTL), Abeta, glutamate (Glu), Abeta + Glu and sulbactam (Sul) + Abeta + Glu groups. For the Sul + Abeta + Glu group, cells were first incubated with sulbactam for 48-h. Then, the culture medium was renewed and Abeta was added to the cultures. After 24-h incubation in the presence of Abeta, the cultures were added with glutamate and incubated for 30 min. For Abeta, Glu, and Abeta + Glu groups, Abeta and glutamate were added to the cultures in the corresponding time points to the Sul + Abeta + Glu group. After the above administration and incubation, the maintenance medium in each group was renewed, and cells were incubated in the renewed maintenance medium for 24-h. Thereafter, cells were harvested for the below assays. Each group included 2–3 wells in each assay and a minimum of five times of independent assays (*n* ≥ 5) were performed.

### CCK8 assay

The formazan dye of the CCK8 solution generated by the dehydrogenase in living cells is directly proportional to the number of viable cells. CCK8 (Cat. No. HY-K0301, MCE, China) solution was added to each well in the volume of 10% maintenance medium. After 2-h incubation, the absorbance of each well was measured at 450 nm by a microplate reader.

### LDH assay

LDH in the cytoplasm is released when the cellular membrane is compromised. The concentration of LDH in the culture medium is proportional to the number of dead cells in cell cultures. Briefly, the supernatant from each well was transferred into a 96-well plate correspondingly. Each well was triplicate. The LDH substrate (Ser. No. A0202-1-2, Nanjing Jiancheng Bioengineering, China) was added according to the protocols and mixed with supernatant thoroughly without bubbles. The supernatant was incubated at 37 °C for 15 min and the stop solution was added. The absorbance of each well was measured at 450 nm by a microplate reader.

### Hoechst–propidium iodide double stains

Neuronal death induced by Abeta and glutamate was analyzed by Hoechst 33258 (HO, Pro. No. 14530, Sigma, USA) and propidium iodide (PI, Pro. No. P4170, Sigma, USA) double stains. Cultures were carefully washed twice with sterile PBS and stained in situ with HO and PI at the same concentration of 10 μg/ml at 37 °C for 15 min. Viable cells were stained nuclei as dark blue (weak HO positive). Dead cells appeared in nuclei as bright blue (intensive HO positive) or red (PI positive). Images were captured with an inverted microscope camera (ZEISS Axiocam 503 color, Germany). Neurons were identified and distinguished from the glial cells by their cell bodies and neurites based on the merged images of bright fields and fluorescent images. At least three views for each well were captured. The cell counting and calculation of neuronal death rate were performed by an investigator who was blinded to the experimental design and grouping.

### Immunofluorescence staining

Primary neuron-astrocyte co-cultures were rinsed with 0.01 M PBS and fixed with 4% paraformaldehyde for 30 min at room temperature, and then the fixed cells were rinsed with 0.01 M PBS. Cells were permeabilized with 0.3% Triton X-100 for 15 min at room temperature and rinsed with 0.01 M PBS. Non-specific bindings were blocked with 5% bovine serum albumin (Cat. No. 4240GR500, BioFroxx, China) at 37 °C for one hour. Cells were incubated with primary antibodies against BCL2, BAX, CCP3, GLT1, glial fibrillary acidic protein (GFAP), or microtubule-associated protein 2 (MAP2) (Table 1) at 4 °C overnight. Cells were rinsed with 0.01 M PBS and incubated with secondary antibodies (Table 1) at 37 °C for one hour. Images were captured with an inverted microscope camera (ZEISS Axiocam 503 color, Germany). At least three views for each well were imaged. The fluorescent density and co-localization were measured and analyzed with the mean gray value and Pearson’s *R*-value by ImageJ. The analysis was performed by an investigator who was blinded to the experimental design and grouping.

**Table 1:** List of antibodies used in this study.

Antibodies	Host	Type	Manufacturer	Cat. no.	Dilution
Anti-BAX	Mouse	Monoclonal	Proteintech	60267-1-lg	1:200 (IF) 1:1000 (WB)
Anti-BCL2	Rabbit	Polyclonal	Immunoway	YT0470	1:200 (IF) 1:1000 (WB)
Anti-Cleaved Caspase 3	Rabbit	Polyclonal	Wanleibio	WL02117	1:200 (IF) 1:500 (WB)
Anti-GLT1	Guinea pig	Polyclonal	Sigma, Millipore	AB1783	1:200 (IF) 1:1000 (WB)
Anti-GAPDH	Mouse	Monoclonal	Proteintech	60004-1-Ig	1:1000 (WB)
Anti-GFAP	Rabbit	Polyclonal	Proteintech	16825-1-AP	1:500 (IF)
Anti-MAP2	Rabbit	Polyclonal	Proteintech	17490-1-AP	1:500 (IF)
CoreLite 488 conjugated goat anti-mouse IgG	Goat	IgG	Proteintech	SA00013-1	1:500 (IF)
CoreLite 488 conjugated goat anti-rabbit IgG	Goat	IgG	Proteintech	SA00013-2	1:500 (IF)
CoreLite 594 conjugated goat anti-mouse IgG	Goat	IgG	Proteintech	SA00013-3	1:500 (IF)
CoreLite 594 conjugated goat anti-rabbit IgG	Goat	IgG	Proteintech	SA00013-4	1:500 (IF)
Horseradish peroxidase-conjugated affinipure goat anti-mouse IgG(H + L)	Goat	IgG	Proteintech	SA00001-1	1:1000 (WB)
Horseradish peroxidase-conjugated affinipure goat anti-rabbit IgG(H + L)	Goat	IgG	Proteintech	SA00001-2	1:1000 (WB)
Biotin-labeled anti-guinea-pig immunoglobulin IgG	Biotin	IgG	KPL	16-17-06	1:1000 (WB)
Horseradish peroxidase-conjugated streptavidin	Horseradish peroxidase	Streptavidin	KPL	43-4323	1:1000 (WB)

*IF* immunofluorescence, *WB* western blotting.

### Western blotting

Cells were collected with 1000 μl pipette tip by triturating them off the well and cells from different wells in each group were combined. Total proteins were extracted in RIPA lysis buffer (Cat. No. R0010, Solarbio, China) containing protease inhibitors (Cat. No. BC3710, Solarbio, China). The protein concentration was assayed with the BCA method (Cat. No. PC0020, Solarbio, China). Twenty micrograms of protein of each sample mixed with loading buffer were loaded in each lane. The samples were electrophoresed in 12% SDS–polyacrylamide gel and transferred to polyvinylidene difluoride membranes (Cat. No. 03010040001, Roche, USA). The nonspecific bindings were blocked in 5% bovine serum albumin (Cat. No. 4240GR500, BioFroxx, China) mixed in distilled water and incubated at 37 °C for 1 h. The membranes were then incubated with primary antibodies against BCL2, BAX, CCP3, GLT1, and GAPDH (Table 1) at 4 °C overnight. The membranes were washed with TBST and incubated with secondary antibodies (Table 1) at room temperature for one hour. For GLT1, the membranes were additionally incubated with horseradish peroxidase-conjugated streptavidin (Table 1) at room temperature for one hour. The immunoreactivities were detected by Omni-ECL Efficient Light Chemiluminescence Kit (Cat. No. SQ203, Epizyme Biotech, China) and scanned with Amersham Imager 600 (UK). ImageJ was used to quantify band signals into integrated density.

### Statistical analysis

After data collection, the post-hoc power analysis was carried out with G*Power 3.1.9.7, and all power values were >0.98 in all experiments. All statistical analysis was performed using GraphPad Prism 8.2.1. The normality was examined by Shapiro–Wilk test and all data passed the test and thus was presented as mean ± standard deviation. The homogeneity of variance was assessed by the Brown–Forsythe test. Data met equal variance (CCK8 and LDH assays, HO–PI double stains, western blotting for BAX/BCL2, GLT1 expressions, and immunofluorescence staining for CCP3, GLT1, and GFAP expressions) was analyzed with one-way ANOVA and followed by multiple comparisons with Tuckey test. Otherwise, data (western blotting for CCP3 expression and immunofluorescence staining for BAX/BCL2, GLT1, and MAP2 expressions) was analyzed with the Welch ANOVA test and followed by multiple comparisons with the Dunnet T3 test. *p* < 0.05 was considered to be statistically significant.

### Supplementary information

Original Western Blot Bands

## Data Availability

The data supporting the findings of the study are available from the corresponding author upon reasonable request.
