# Supplementary figures and images for: Sulbactam protects neurons against double neurotoxicity of amyloid beta and glutamate load by upregulating glial glutamate transporter 1
Source: Cell Death Discov. 2024 Feb 6;10:64. doi: 10.1038/s41420-024-01827-5 (PMC10847450; doi:10.1038/s41420-024-01827-5)

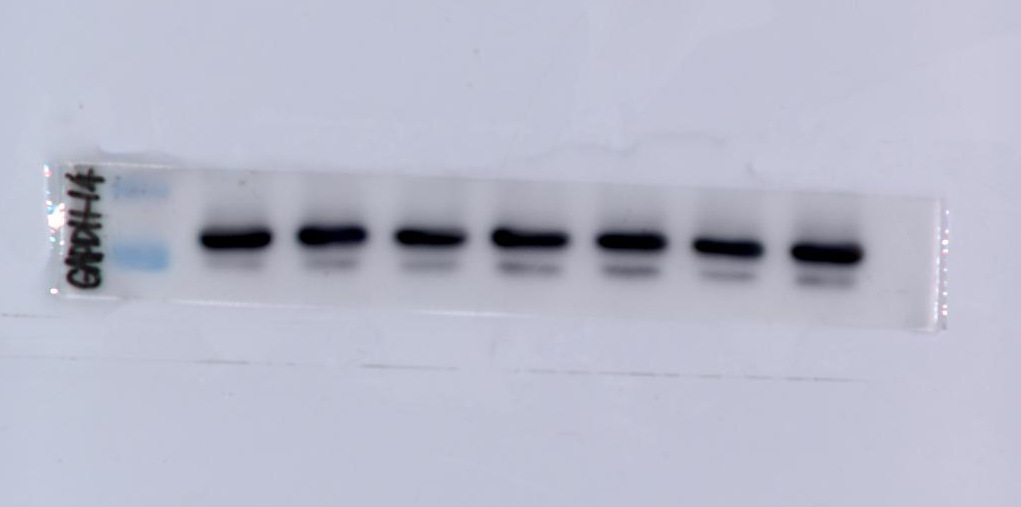

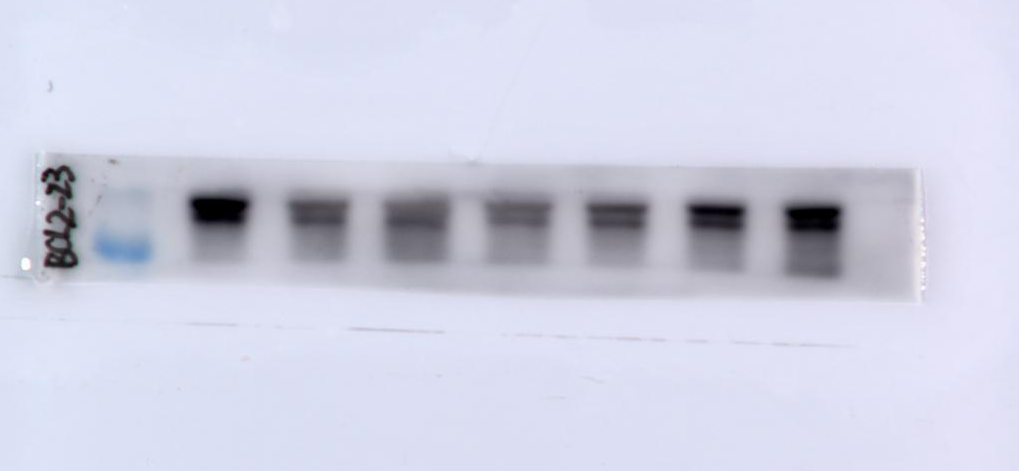

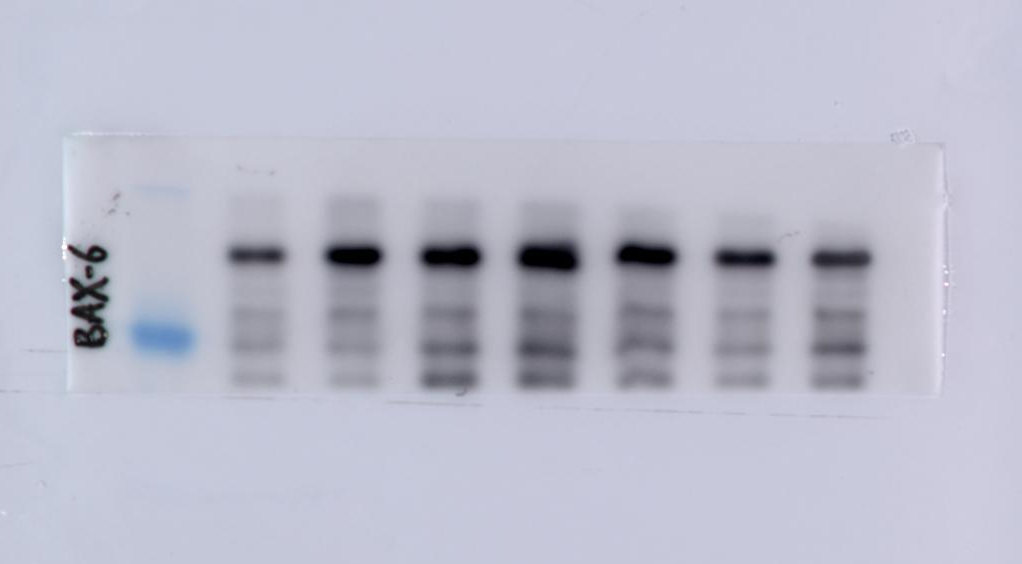


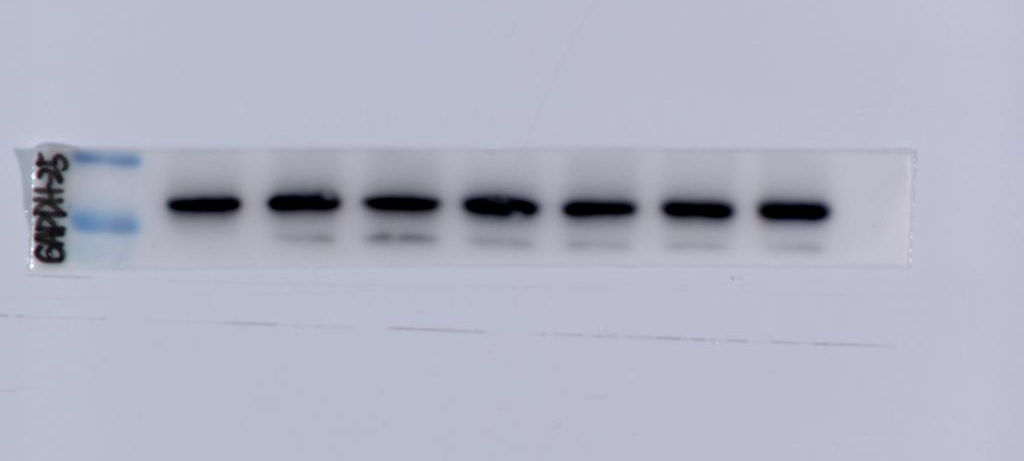


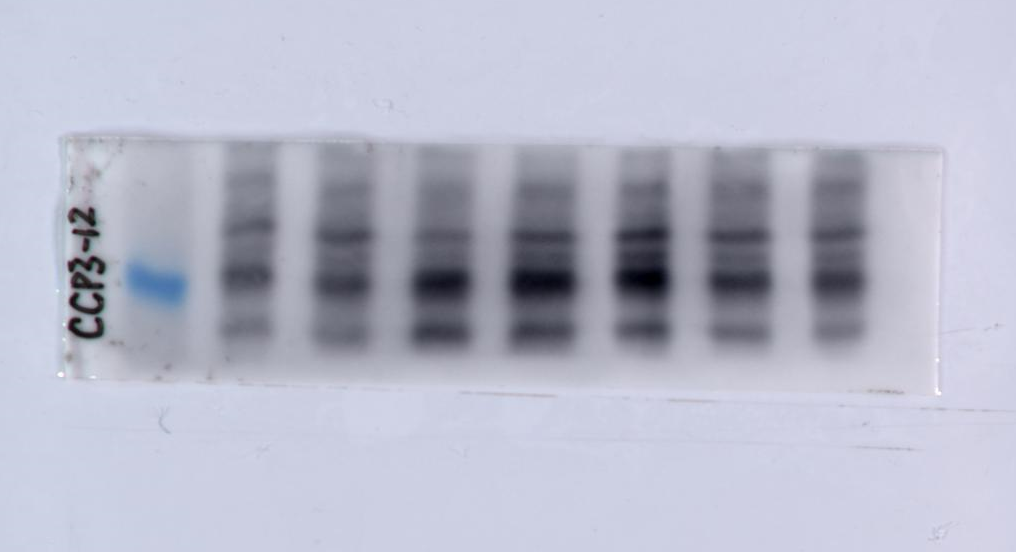


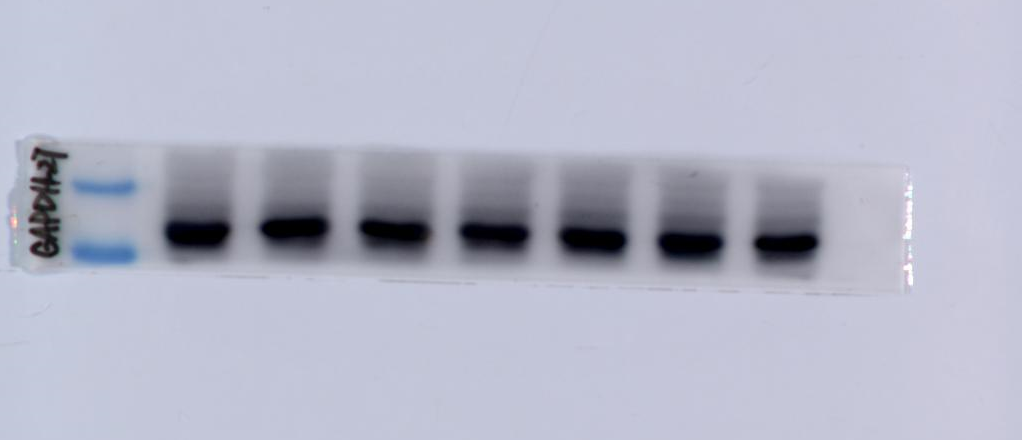

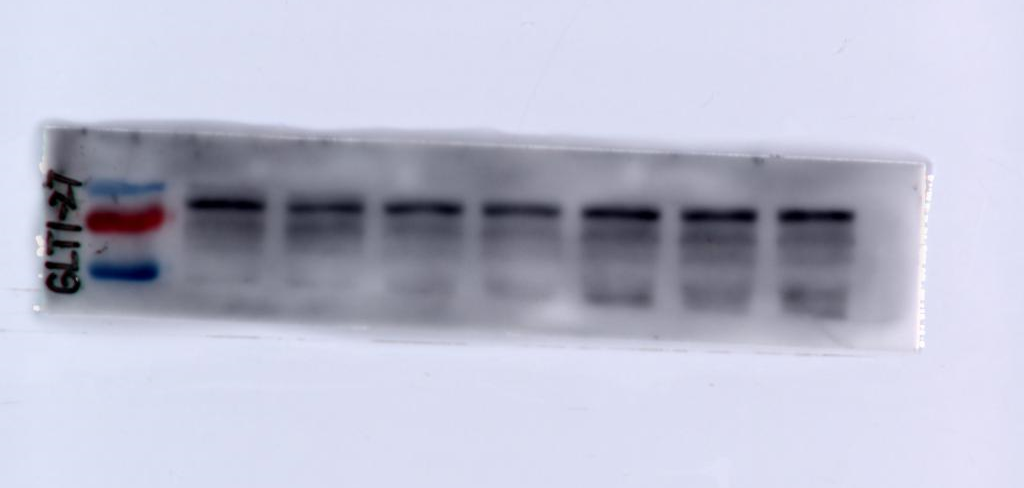

Supplement: Supplementary file 1 — Original Western Blot Bands [file 41420_2024_1827_MOESM1_ESM.docx]
